# Association of Home Based Primary Care Enrollment with Social Determinants of Health for Older Veterans

**DOI:** 10.1093/geroni/igab046.3306

**Published:** 2021-12-17

**Authors:** Anna-Rae Montano, Augustus Ge, Christopher Halladay, Samuel Edwards, James Rudolph, Portia Cornell

**Affiliations:** 1 Providence VA Medical Center, Providence, Rhode Island, United States; 2 Department of Veterans Affairs, Providence, Rhode Island, United States; 3 VA Portland Health Care System, Portland, Oregon, United States

## Abstract

The Veterans Administration (VA) Home-based Primary Care (HBPC) program provides comprehensive primary care to older Veterans with multiple chronic conditions who may be at risk of adverse health outcomes due to their social determinants of health. Area Deprivation Index (ADI) can be used as a surrogate measure of a Veteran’s social needs. The objective of this study was to estimate the effect of neighborhood disadvantage, as measured by ADI, on HBPC enrollment for a sample of older Veterans. We estimated a linear multivariate model in which the exposure was ADI and the outcome was enrollment in HBPC. Controls included clinical and demographic characteristics. In a final sample of 12,005,453 observations (total Veteran months) on 353,485 individual Veterans, 18.4% lived in high-deprivation neighborhoods (ADI greater than or equal to 80). Mean monthly probability of new HBPC enrollment was 0.0061. Controlling for clinical characteristics, housing instability, and distance from the medical center, Veterans residing in high-deprivation neighborhoods were 1.4% to 14.8% less likely to enroll in HBPC, though the association was not statistically significant. The VA HBPC program provides beneficial comprehensive, primary care services to Veterans at risk of poor health outcomes. However, a Veteran’s social determinants of health could prevent enrollment. More research is needed to determine the relationship between Veterans’ social needs and HBPC enrollment.

